# Impacts of chemical gradients on microbial community structure

**DOI:** 10.1038/ismej.2016.175

**Published:** 2017-01-17

**Authors:** Jianwei Chen, Anna Hanke, Halina E Tegetmeyer, Ines Kattelmann, Ritin Sharma, Emmo Hamann, Theresa Hargesheimer, Beate Kraft, Sabine Lenk, Jeanine S Geelhoed, Robert L Hettich, Marc Strous

**Affiliations:** 1 Department of Geoscience, University of Calgary, Calgary, AB, Canada; 2 Max Planck Institute for Marine Microbiology, Bremen, Germany; 3 Institute for Genome Research and Systems Biology, Center for Biotechnology, Bielefeld University, Bielefeld, Germany; 4 Chemical Science Division, Oak Ridge National Laboratory, Oak Ridge, TN, USA; 5 UT-ORNL Graduate School of Genome Science and Technology, University of Tennessee, Knoxville, TN, USA; 6 Department of Molecular Oncology, The Moffitt Cancer Center and Research Institute, Tampa, FL, USA; 7 Nordic Center for Earth Evolution, University of Southern Denmark, Odense, Denmark; 8 NIOZ Royal Netherlands Institute for Sea Research, Yerseke, The Netherlands

## Abstract

Succession of redox processes is sometimes assumed to define a basic microbial community structure for ecosystems with oxygen gradients. In this paradigm, aerobic respiration, denitrification, fermentation and sulfate reduction proceed in a thermodynamically determined order, known as the ‘redox tower'. Here, we investigated whether redox sorting of microbial processes explains microbial community structure at low-oxygen concentrations. We subjected a diverse microbial community sampled from a coastal marine sediment to 100 days of tidal cycling in a laboratory chemostat. Oxygen gradients (both in space and time) led to the assembly of a microbial community dominated by populations that each performed aerobic and anaerobic metabolism in parallel. This was shown by metagenomics, transcriptomics, proteomics and stable isotope incubations. Effective oxygen consumption combined with the formation of microaggregates sustained the activity of oxygen-sensitive anaerobic enzymes, leading to braiding of unsorted redox processes, within and between populations. Analyses of available metagenomic data sets indicated that the same ecological strategies might also be successful in some natural ecosystems.

## Introduction

In biogeochemistry, observation of chemical gradients in poorly mixed environments led to the development of a theorem known as the microbial redox ‘tower', ‘ladder' or ‘cascade'. This theorem holds that a succession (in space or time) of ecological guilds of prokaryotes consumes electron acceptors in a thermodynamically determined order (‘thermodynamic sorting' Richards, 1965; Orcutt *et al.*, 2011). Aerobic bacteria (E_0_′+0.82 V) first consume oxygen until it is depleted. Subsequently, denitrifying bacteria convert nitrate to nitrogen gas (average E_0_′+0.75 V). Reduction of manganese and iron oxides is next in line, followed by sulfate reduction (E_0_′−0.22 V) and finally respiration of carbon dioxide to methane (E_0_′−0.24 V). Fermentation is usually placed between denitrification and sulfate reduction (Fenchel and Jørgensen, 1977).

In theory, the redox tower is also an attractive model to explain community structure in microbial ecology. Natural microbial communities are complex and their study is challenging, even with modern metagenomics approaches (Newman and Banfield, 2002; Oremland *et al.*, 2005; Woodcock *et al.*, 2007; Little *et al.*, 2008). We are in need of organizing principles explaining microbial community structure and the redox tower might constitute such a principle. If nature would select for microbial communities that convert the available resources with the production of the highest possible amount of biomass, redox tower behavior would be the outcome (Rodriguez *et al.*, 2008; Temudo *et al.*, 2008). If nature would select for microbial communities that convert the available resources as quickly as possible, loss of thermodynamic sorting may result. From a thermodynamic perspective, the first scenario is consistent with Prigogine's theorem of the non-equilibrium steady state and the second is consistent with Ziegler's principle (Bordel, 2010). Because metabolism is by definition outside thermodynamic equilibrium, thermodynamic sorting of redox processes cannot be deduced from first principles and it does not need to be true in general. Thus, thermodynamic sorting may be a possible outcome of community assembly that applies when certain conditions are met.

In microbiology, it has long been known that at least microbial strains isolated in pure culture commonly abide to the redox tower, which is easily explained by inhibition of low redox potential processes by high potential electron acceptors. However, non-redox tower behavior has also been observed. For example, denitrification was observed in the presence of oxygen (Takaya *et al.*, 2003; Jensen *et al.*, 2009; Gao *et al.*, 2010), sulfate reduction in the presence of nitrate (Dalsgaard and Bak, 1994; Canfield *et al.*, 2010), methanogenesis in the presence of sulfate (Kuivila *et al.*, 1990) and fermentation in the presence of oxygen (Thomson *et al.*, 2005). The latter observation is known as the ‘Crabtree effect' in industrial yeast production and the ‘Warburg effect' in cancer biology (Crabtree 1928; Warburg 1956; Diaz-Ruiz *et al.*, 2011).

Unfortunately, many studies do not report the degree of aggregation of cells, for example, while reporting so-called ‘aerobic denitrification' (Robertson *et al.*, 1989; Chen *et al.*, 2003; Takaya *et al.*, 2003; Khardenavis *et al.*, 2007; Su *et al.*, 2015). Diffusion of oxygen towards single bacterial cells is so effective that with the reported cellular respiration rates, a single aerobic cell can never deplete all the oxygen in its immediate surroundings, unless the bulk oxygen concentration is in the low nanomolar range (Schulz and Jørgensen 2001; Stolper *et al.*, 2010). However, a microaggregate (diameter >5 μm) of tens to hundreds aerobic bacterial cells can effectively respire all oxygen in its immediate surroundings at bulk oxygen concentrations of ~20 μm (~10% air saturation) and this could in theory explain apparently non-redox tower behavior.

The present study addresses how spatio-temporal chemical gradients shape microbial community structure at low (<20 μm) oxygen and nitrate concentrations. If the redox tower would apply, aerobic and anaerobic processes would occur in separate (in space or time) microcompartments. If not, these processes would occur in parallel in the same compartment. The concept would also apply to gradients of nitrate, sulfate and so on.

The research question was addressed experimentally by sampling a microbial community from a tidal flat sediment naturally exposed to oxygen gradients in space and time. The sampled community was incubated in a laboratory chemostat with well-defined spatio-temporal oxygen gradients. During 100 days, a simple, more or less stable microbial community had assembled from the much more complex source community. See also Yan *et al.* (2012), describing a similar approach. A combination of independent methods showed that this simplified community did not abide to the microbial redox tower. Instead, the metabolism was ‘braided', with each population performing a combination of aerobic and anaerobic metabolism and exchange of partially converted substrates between populations. Future study will show to what extent this observation can be generalized (see for example, Hawley *et al.*, 2014). However, active transcription of key genes characteristic of the observed braided metabolism in two natural ecosystems with oxygen gradients, confirms (Canfield *et al.*, 2010) and expands previous results. Our study indicated that microbial communities may convert available resources as quickly as possible, increasing resilience at the price of reduced productivity.

## Materials and methods

### Sampling, cell extraction, continuous cultivation and online mass spectrometry

Sediment samples for incubations were taken from an intertidal sand flat in the central German Wadden Sea (N53°44.151′, E007°41.943′) on 1 November 2011. Bacteria were extracted from the sediments and cultivated in continuous culture for 100 days as previously described (Hanke *et al.*, 2014). Briefly, the 2.8 l culture vessel was mixed by recirculation of gas (4 l min^−1^) from the top of the vessel to the bottom, via a sintered glass filter. The filter-sterile (0.2 μm, Sartopore MidiCaps, Sartorius, Göttingen, Germany) medium was continuously supplied at a dilution rate of 0.26 per day and contained (mm): NaNO_2_ 20.0, NaNO_3_ 1.0, FeSO_4_ 0.01, KH_2_PO_4_ 0.50, CaCl_2_ 0.0013, glucose 6.0, acetate 3.1, glutamate 1.1, aspartate 1.5, alanine 1.5, serine 0.80, tyrosine 0.51, histidine 0.12 and methionine 0.25, 33.4 g l^−1^ Red Sea Salt (Aquaristic.net) and 0.2 ml l^−1^ trace element solution (Hanke *et al.*, 2014). The pH and temperature were controlled at 8.0 and 25 °C, respectively. Pure oxygen was supplied at 20 ml min^−1^ for 5 min, once every 12 h (one tidal cycle). Off gas was led to a GAM 400 mass spectrometer (In Process Instruments, Bremen, Germany) for online detection of O_2_, Ar, N_2_ and N_2_O after replacing the nitrite in the medium with its ^15^N-labeled form.

### Incubation of suspended cells

Incubations were performed in the dark in 200 ml serum flasks stirred at 300 r.p.m. with a magnetic stirrer bar and fitted with optical oxygen sensors (FSO2-4, Pyroscience, Aachen, Germany). A final concentration of 1.0 mm ^15^N-nitrite or nitrate and 0.30 g HEPES (4-(2-hydroxyethyl)-1-piperazineethanesulfonic acid) were added to the medium (composition, see above). The oxygen concentration was set by adjusting the O_2_/Ar ratio in the headspace, and validated mass spectrometrically (GAM 400, In Process Instrument, Bremen, Germany). The cells sampled from the culture were injected through a 5 μm pore size syringe filter. The gas and liquid samples were taken once every 20–30 min for 3 h. In control experiments for nitrate inhibition of aerobic respiration, seawater was injected instead of nitrate.

### Chemical analysis

The presence or absence of nitrite and nitrate was determined with Quantofix test strips (0–80 mg l^−1^ NO_2_^−^, 0–500 mg l^−1^ NO_3_^−^, Merck, Germany). Nitrite, ammonium and protein and sulfur concentrations were quantified as previously described (Lowry *et al.*, 1951; Van Eck, 1966; Kamyshny *et al.*, 2006). Fatty acids were quantified with high-performance liquid chromatography (Albert and Martens, 1997). The dissolved oxygen was measured using an optical oxygen meter (FSO2-4, PyroScience). Particulate organic carbon was determined with cuvette-tests (Hach Lange GmbH, Düsseldorf, Germany) in a Thermostat LT200 and a DR3900 photometer (Hach Lange GmbH). The protein content of cells was calculated from protein and particulate organic carbon measurements.

### Genomics, transcriptomics, proteomics and *in silico* procedures

**Figure 1:**
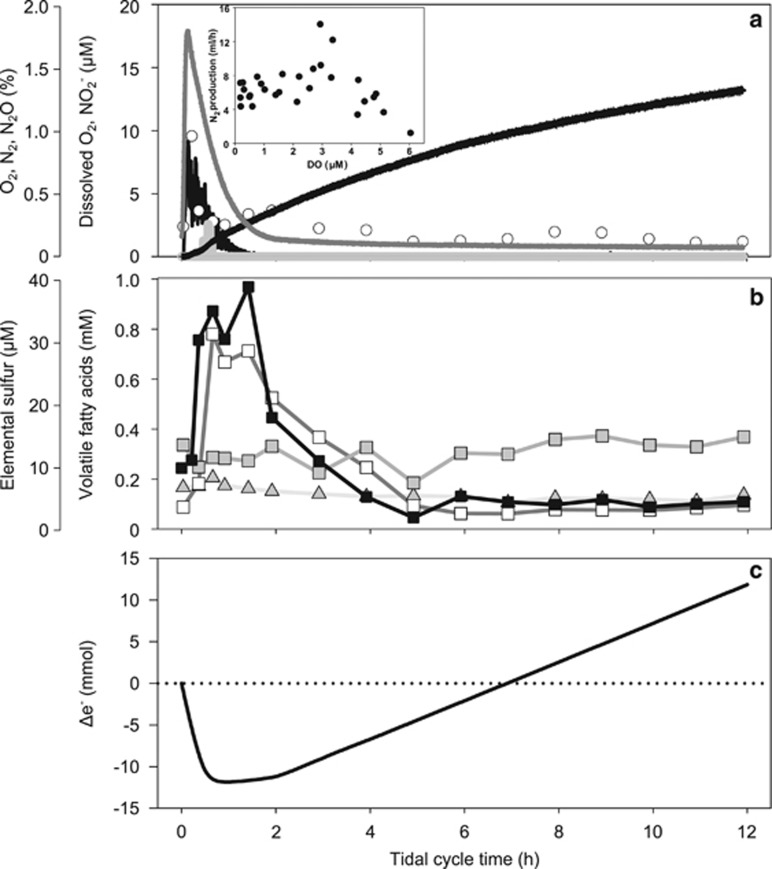
Concentrations of substrates and products during tidal cycling. (**a**) Concentrations of 15-NO_2_^−^ (circles) and dissolved O_2_ (black line showing transient accumulation) in the culture, and headspace concentrations of O_2_ (gray line), ^30^N_2_ (black line showing continuous accumulation) and ^46^N_2_O (light gray line) during tidal cycling on day 87. The inset shows the denitrification rate as a function of dissolved oxygen concentration. (**b**) Concentrations of formate (open squares), elemental sulfur (solid squares), acetate (gray squares) and succinate (triangles) during the same cycle. (**c**) Cumulative electron balance during the tidal cycle. Δe is the difference between electrons supplied in the form of glucose, acetate and amino acids, and electrons accepted by the supplied nitrate, nitrite and oxygen.

For 16S ribosomal RNA (rRNA) gene tag sequencing, primers S-D-Arch-0519-a-S-15 and S-D-Bact-0785-b-A-18 covering the v4 region (Klindworth *et al.*, 2013) were used and extended with MIDs of 7 to 10 bp in length for multiplexing. PCR was performed using 25–125 ng template DNA and the Phusion High-Fidelity PCR Master Mix (Finnzymes, Thermo Fisher Scientific, Waltham, MA, USA) with the following protocol: 30 s initial denaturation (98 °C), 30 cycles of 10 s denaturation (98 °C), 30 s primer annealing (66 °C) and 12 s extension (72 °C), and a final extension step at 72 °C for 10 min. The amplicons (approximately 300 bp in length) were purified and pooled, an end-repair step was performed, adaptors for PGM sequencing were ligated and the resulting ion torrent libraries were sequenced on the PGM platform using 400 bp read chemistry. A total of 14 samples taken at different time points were sequenced yielding 729 581 reads. After demultiplexing and filtering with trimmomatic (Bolger *et al.*, 2014; quality >20) and mothur (Schloss *et al.*, 2009; bdiffs=1, pdiffs=2, minlength=250, maxlength=350, maxambig=0, maxhomop=8), 349 170 reads remained. These were classified to the assembled 16S rRNA gene sequences, one for each bin, with USEARCH (Edgar, 2010) at 92% and 97% identity thresholds. Number of Operational Taxonomic Units (OTUs) were determined for each sample and each clade by clustering with USEARCH at 97% identity. The RNA samples were fixed immediately with RNAlater (Ambion, Austin, TX, USA). Two-milliliter cultures were used to extract DNA or RNA (Zhou *et al.*, 1996; Smith *et al.*, 2007). The isolated DNA and RNA were sequenced with the Ion Personal Genome Machine (PGM) System (Thermo Fisher Scientific, San Francisco, CA, USA) on 316 and 318 chips, respectively. The combined reads of all three DNA samples were assembled from the sff files generated by the Torrent Suite software 2.0.1 with the GS De Novo Assembler 2.6 (454 Life Sciences, Branford, CT, USA default settings for genomic DNA). To recover 16S rRNA genes, an additional assembly was performed (Fan *et al.*, 2012). Briefly, the complementary DNA option was selected during sff files loading, the ‘Minimum overlap identity' was set to 99%, and ‘Extend low depth overlaps' as well as ‘Reads limited to one contig' were selected. Contigs were binned using tetranucleotides with MetaWatt with its most relaxed settings, as previously described (Strous *et al.*, 2012). Binning yielded four bins, representing three of the five abundant clades (for one of the clades, two bins were detected, see Supplementary Figure 1). Detection and phylogeny of 16S rRNA genes was performed as previously described (Hanke *et al.*, 2014). Abundances of all clades were estimated on the basis of sequenced DNA and RNA by mapping sequencing reads to the assembled contigs with BBMap (http://sourceforge.net/projects/bbmap/, k 13, minid 0.6). For the two clades that assembled poorly (clades E and F), genomes of closely related bacteria were used as the template for mapping (*D. salexigens*, Genbank CP001649 and BioProject PRJNA246767/Bin A). The assembled contigs were annotated separately for each bin with prokka (Seemann, 2014). Transcriptional per-gene activities were computed for all predicted open reading frames (ORFs) from the mapped transcriptomes by dividing (number of reads mapped to ORF/ORF length) by (total number reads mapped to all ORFs in bin/total length of all ORFs in bin). This way, a transcriptional activity of 1.0 corresponded to the average transcriptional activity. For Bin D (*Vibrionales*), less conserved regions of some key genes were assembled incompletely. For those genes, gene completeness was validated independent of assembly, by performing blastx of all reads (both transcriptomes and metagenomes) against the genome of a related reference organism (*Vibrio tubiashii*). RNA was extracted from the culture at two different time points during tidal cycling shown in Figure 1, RNA1 at 0.3 h, at the peak of the oxygen profile and RNA2 at 2 h, the base of the oxygen profile, and from anoxic (RNA3) and oxic (RNA4) incubations of filtered cells shown in Figure 4, at 0.0 mm and 0.3 mm O_2_, respectively.

All sequencing reads generated in this study, including amplicon libraries and transcriptomes, as well as the assembled contigs are available at the NCBI (PRJNA255238).

Proteomic analyses of two technical replicates was performed as previously described (Hanke *et al.*, 2014). Population abundances estimated from the proteomic results were estimated by counting unambiguously identified peptides for each bin. Sequencing reads from metagenomes and transcriptomes from the Chilean oxygen minimum zone (Stewart *et al.* 2012) (www.ncbi.nlm.nih.gov/Traces/sra/?study=SRP003331) and the Janssand tidal flat (Sequence Read Archive SRP021900) were translated in six frames and scanned for key functional genes with Hidden Markov Models constructed from aligned reference amino acid sequences, one for each gene (*pflAB*, *bd*-I, *bd*-II, the heme-copper oxidase superfamily, *dsrAB*, *nirS* and *rpoBC*) with hmmscan (version 3.0) (Eddy, 2008) with an e-value cut-off of 1e−10. Read counts for each functional gene were normalized against counts for *rpoBC*. Gene abundances were inferred from metagenomes, gene activities from transcriptomes.

### Fluorescence *in situ* hybridization

FISH (fluorescence *in situ* hybridization) and catalyzed reporter deposition-FISH (CARD-FISH) were performed as previously described (Llobet-Brossa *et al.*, 1998; Pernthaler *et al.*, 2004). The probes and formamide concentrations used are shown in Supplementary Table 1.

### Substrate consumption and supply for suspended cells and microaggregates

The oxygen flux *J* towards a single cell or aggregate of cells is (Fenchel *et al.*, 2012):


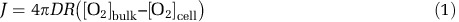


With *D*, the diffusion coefficient (10^−9^ m^2^ s^−1^), *R*, the radius of a static boundary layer surrounding the cell or aggregate (>10^−5^ m, the Kologorov scale in turbulent systems), [O_2_]_bulk_, the oxygen concentration as measured in the liquid and [O_2_]_cell_, the concentration of oxygen at the cell or aggregate surface. When the oxygen is depleted at the cell surface ([O_2_]_cell_=0), the oxygen uptake rate of the cell or aggregate should be equal to *J*. The average oxygen uptake rate of the cells in the continuous culture was at most 20 mmol g^−1^ protein per hour (Figure 4e), or, depending on the cell diameter, at most 25 fmol per cell per day for spherical cells 1 μm wide.

### Stoichiometric modeling of metabolism

Stoichiometric modeling is described in the Supplementary Online Information.

## Results

Microbial cells were extracted from the upper 2 cm of sediments collected from the Wadden Sea, one of the largest intertidal systems of the temperate world. These sediments are naturally exposed to oxygen at high tide and become largely anoxic during low tide (Billerbeck *et al.*, 2006). The extracted cells were incubated in a laboratory chemostat. Oxygen was supplied twice daily and remained available for approximately 2 h after supply (Figure 1), simulating the natural temporal oxygen dynamics. Artificial seawater containing nitrate (1 mm), nitrite (20 mm) and a mixture of carbon compounds was supplied and removed continuously, so that the actual substrate concentrations in the culture always remained low (for example, <5 μm nitrite, <10 μm nitrate, <0.4 mm acetate). Nitrite, rather than nitrate, was supplied as the main substrate for denitrification because the sampled microbial community stoichiometrically converted nitrate to nitrite, leading to high (>1 mm), potentially toxic, concentrations of nitrite, whereas, when nitrite was supplied, it was always almost fully converted to gaseous products. In marine sediments, nitrification and denitrification are generally tightly coupled so it was unknown whether nitrite or nitrate was the main substrate for denitrification *in situ*. The supplied mixture of carbon compounds consisted of glucose (~50% of the carbon supplied), acetate (~10%) and seven different amino acids (~40%). These monomers represented the main classes of molecules set free in approximately the same ratio during the hydrolysis of detritus, the primary carbon and energy source *in situ*. Because the electron donors were supplied continuously and oxygen was only available during two 2 h periods, this led to an imbalance in the relative availability of electron donors and acceptors (Figure 1c). The supplied carbon and energy sources donors were consumed (almost) completely at all times, indicating that the microbial community must have stored part of them during the anoxic period of the cycle to sustain aerobic metabolism during the oxic part.

**Figure 2:**
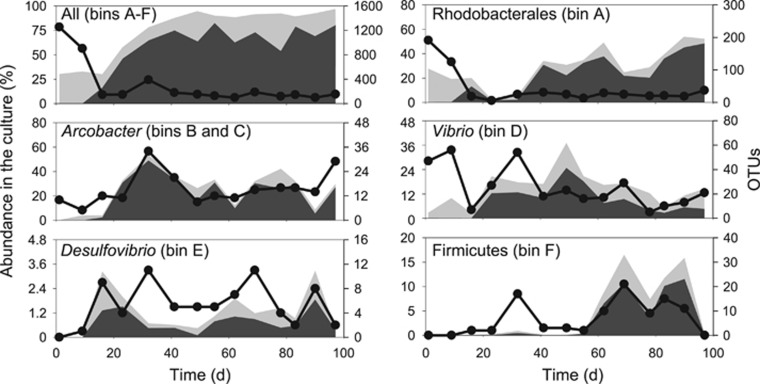
Outcome of microbial community assembly in the chemostat. Abundances in 16S rRNA gene tag libraries of the dominant representative (black areas, 97% identity cut-off) of the five most abundant clades (gray areas, 92% identity cut-off) and number of operational taxonomic units (OTUs) within each clade (black circles, 97% identity cut-off) show selection of clades and of dominant representatives within the clades.

The chemostat was run without external interruption for over 3 months, approximately 37 microbial generations at the dilution rate applied (0.26 per day). During these months, a simple microbial community assembled from the more complex inoculum. 16S rRNA gene amplicon sequencing (Figure 2) revealed that microbial populations representing five different phylogenetic clades became abundant members of the chemostat's microbial community. Relative abundances estimated from 16S rRNA gene amplicon sequencing were validated with FISH microscopy on day 83 (Supplementary Table 2) and were also supported by shotgun metagenenomic sequencing (*n*=3), sequencing of transcriptomes (*n*=4) and proteomics (*n*=1; Supplementary Table 2).

The overall communal metabolism of the microbial community was determined by monitoring concentrations of carbon, nitrogen and sulfur compounds during the chemostat's ‘tidal' cycle (Figure 1). To more precisely quantify nitrogen conversions, we supplied medium with ^15^N-labeled nitrite during these experiments and measured the gaseous products with real time mass spectrometry. The dissolved oxygen concentration of the culture liquid was monitored online with an optical oxygen probe. After oxygen was supplied at the start of a cycle, transient accumulation of the denitrification intermediate nitrous oxide (46-N_2_O) was observed, but nitrogen (30-N_2_) was always the main product of denitrification. Apparently, denitrification to N_2_ proceeded even in the presence of oxygen in the bulk medium, as was reported for the intertidal flat itself (Gao *et al.*, 2010).

‘Tidal' cycling resulted in the transient accumulation of formate (0.8 mm) and elemental sulfur (30 μm) during the oxic periods (Figure 1b). This was surprising, because during this period electron donors were in short supply and production of a reduced compound would not be expected.

To determine the ecological functions of the chemostat's populations, DNA was extracted on days 83, 90, 97 and subjected to shotgun sequencing. Sequencing reads were assembled and binned (Supplementary Figure 1,Supplementary Table 2), resulting in a provisional whole-genome sequence for the dominant representative of three of the five major clades shown in Figure 2. The expression of the predicted genes by each population was determined with proteomics and the effect of the tidal cycling on protein expression was analyzed with transcriptomics (see Supplementary Dataset S1–S10). For all the populations, a complete 16S rRNA gene was assembled that was nearly identical to the 16S rRNA gene amplicon of the dominant representative of each clade (Supplementary Figure 2). The abundance of two populations (clades E and F) was too low to enable meaningful assembly (except for 16S rRNA genes, which presumably had higher copy numbers). For these populations, we used reference genomes with nearly identical 16S rRNA genes (Supplementary Figure 2) to infer gene expression. Even though the match of the reference genomes was likely imperfect, many proteins and transcripts could still be identified for these populations (Supplementary Table 2).

**Figure 3:**
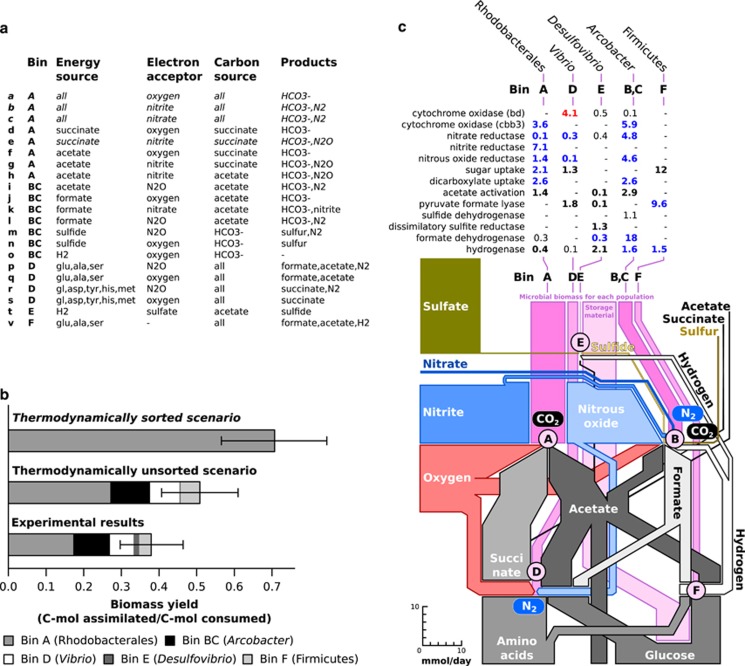
Unraveling of communal metabolism based on gene activities, protein expression and mathematical modeling. (**a**) Metabolic reactions used for metabolic modeling. (**b**) Relative population abundances and overall biomass yield for the thermodynamically sorted (reactions *a*–*c*) and unsorted (reactions *d*–*v*) scenarios compared with the experimental observations. (**c**) Selected gene activities and layout of the ‘braided' communal metabolism predicted by the model. Each population (bins A–F) is shown as a node (pink circles) in the network, with arrows showing the conversion of substrates into products by each population. The conversion of the supplied carbon and energy sources is shown from bottom to top and the conversion of the supplied electron acceptors is shown from left to right. Pink arrows show the amount of biomass produced for each population and the amount of storage materials produced, which was not assigned to any specific population. Width of all arrows is proportional to the calculated elemental flows (mmol C, O, N, S per day, horizontal and vertical scale bar in lower left corner). The table shows transcriptome-inferred gene activities of key metabolic modules for the six abundant populations (bins A–F). Bold numbers indicate that expression was detected experimentally by proteomics. Red and blue numbers indicate upregulation in the presence and absence of oxygen, respectively.

The detected peptides and transcripts suggested that *Vibrio* and Firmicutes (bins D and F, Figure 2) fermented carbon substrates. These populations apparently converted glucose and the amino acids alanine and serine into formate, acetate and hydrogen, as shown by elevated transcriptional activities of glycolysis, alanine dehydrogenase, hydrogen-evolving hydrogenases and pyruvate formate lyase, a glycyl radical enzyme extremely sensitive to oxygen. Gene activities of *Vibrio* and Firmicutes were consistent with the fermentation of other amino acids (also present in the medium) into succinate (see also Figure 1b,Supplementary Dataset). At the same time, *Vibrio* respired oxygen with a cytochrome *bd* terminal oxidase and potentially converted nitrate to nitrite and nitrous oxide to N_2_. Cytochrome *bd* terminal oxidase was also actively expressed by sulfate reducing *Desulfovibrio* (bin E). Fermentation of glucose into formate and acetate generates excess electrons. Most likely, *Vibrio* disposed of these electrons with its respiratory chain, whereas Firmicutes led them toward its hydrogen-evolving hydrogenase, generating H_2_ (see Figure 3c).

Rhodobacterales (bin A) and *Arcobacter* (bins B and C) were inferred to perform denitrification and aerobic respiration. For the latter, both made use of a *cbb*_3_-type terminal oxidase. The two populations appeared to focus on different steps in the denitrification pathway. *Arcobacter* only possessed genes for the reduction of nitrate to nitrite and nitrous oxide to N_2_. For Rhodobacterales, transcriptional activities were highest for genes performing reduction of nitrite to nitrous oxide. Gene activities suggested that both populations oxidized fermentation products such as dicarboxylates (for example, succinate), acetate, formate and hydrogen, but the Rhodobacterales population also appeared to compete for glucose. Consistent with experimental observations (Figure 1b), the *Arcobacter* populations apparently oxidized sulfide incompletely to elemental sulfur, which accumulated in the medium and as a film on the glass wall of the culture vessel.

The overall biomass growth yield of the assembled microbial community decreased from 0.44±0.03 (day 49) to 0.32±0.02 C-mol assimilated/C-mol consumed from day 83 onward. For comparison, growth yields up to ~0.7 C-mol assimilated/C-mol consumed were reported for pure cultures oxidizing glucose with oxygen or nitrate (Heijnen *et al.*, 1992) and yields of 0.3 C-mol/C-mol were reported for denitrifying cultures oxidizing acetate (Strohm *et al.*, 2007).

To assess whether the observed overall biomass yield, relative population abundances and per-population gene expression patterns were consistent with the current understanding of microbial bioenergetics, we created a stoichiometric model of communal metabolism. In this model, all the major populations performed one or more metabolic reactions (Figure 3a, reactions *a*–*v* and Supplementary Dataset S11). All metabolic reactions were the sum of a catabolic reaction and an anabolic reaction. For example, in reaction *d*, the catabolic reaction was the aerobic respiration of succinate and the anabolic reaction was the assimilation of succinate into biomass. The biomass yield (amount of carbon substrate assimilated divided by the total amount of carbon substrate converted) was estimated separately for each reaction according to Heijnen *et al.* (1992). Reactions *a*–*c* described the canonical respiration of the complete carbon mixture (including glucose) to CO_2_ with oxygen, nitrite and nitrate respectively. On the basis of gene expression patterns, these reactions were assigned to Rhodobacterales. The model was solved by adjusting the rates of all metabolic reactions until the modeled overall substrate conversions matched the experimentally observed overall conversions, including growth. In the solution that most closely approximated the experimental results (Figure 3b), the rates of reactions *a*–*c* were zero. This suggested that *Vibrio* and Firmicutes fermented almost all the high quality carbon substrates (glucose and amino acids) and that the three other populations used the fermentation products for aerobic respiration, denitrification, sulfate reduction and assimilation. The complex substrate conversions of the ‘braided' metabolism according to the model are shown in Figure 3c.

**Figure 4:**
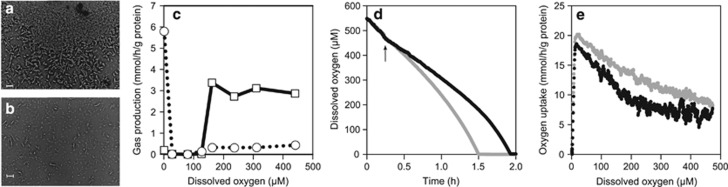
Aerobic denitrification by suspended bacteria. (**a**) Aggregated cells sampled from the culture. (**b**) Suspended cells obtained by filtration. (**c**) Production of nitrogen (circles) and nitrous oxide (squares) as a function of oxygen concentration. (**d**) Inhibition of oxygen consumption after injection of 0.8 mm nitrate (arrow, black line) compared to control (gray line). (**e**) Inhibition of oxygen consumption as a function of oxygen concentration in the presence (black) and absence (gray) of nitrate. Scale bar, 5 μm.

According to Figure 3c, fermentation of carbon substrates occurred before respiration and denitrification proceeded in parallel with aerobic respiration. This suggested the activity of known oxygen-sensitive enzymes such as pyruvate formate lyase and nitrous oxide reductase in the presence of oxygen. Microscopic investigation indicated the presence of microaggregates in the chemostat (Figure 4a). To investigate whether these microaggregates provided protection to oxygen-sensitive enzyme systems, the culture was filtered through a 5 μm membrane filter. The filtrate, containing only suspended bacterial cells (Figure 4b), was incubated with the carbon substrates and ^15^N-labeled nitrite, at different oxygen concentrations. Transcriptomic analysis of the suspended and aggregated fractions showed that among the five most abundant clades, the *Arcobacter* populations were ~2 × less abundant in the cell suspensions, *Vibrio* populations were ~3 × more abundant in the unfiltered samples and the other clades showed no difference (Supplementary Table 2). With suspended cells, complete denitrification was no longer observed in the presence of oxygen and was completely abolished at oxygen concentrations between 25 μm and 125 μm (Figure 4c). Interestingly, above 125 μm O_2_ (~60% air saturation), denitrification recovered, but with nitrous oxide rather than nitrogen as the end product. We also showed that oxygen consumption was inhibited in the presence of nitrate (Figure 4d). Oxygen consumption was also inhibited at high oxygen concentration (Figure 4e), as might be expected for high affinity *cbb*_*3*_-type terminal oxidases (Pitcher and Watmough, 2004).

Finally, we investigated whether key genes, characteristic of the braided, unsorted communal metabolism of Figure 3c were also active in two natural oxygen-limited ecosystems. Available metagenomes and metatranscriptomes from the Chilean oxygen minimum zone (Canfield *et al.*, 2010; Stewart *et al.*, 2012) and our sampling site, an intertidal flat in the Wadden Sea (Kraft *et al.*, 2014), were inspected for the presence and activity of heme-copper terminal oxidases (including the *cbb*_*3*_ type), as a marker gene for aerobic respiration, cytochrome *cd* nitrite reductase (*nirS*), as a marker gene for denitrification, pyruvate formate lyase (*pflAB*), as a marker gene for fermentation, dissimilatory sulfate reductase (*dsrAB*), as a marker gene for sulfate reduction and the *bd-*type terminal oxidase, as a marker gene for the elimination of oxygen to support fermentation or sulfate reduction. Numbers of metagenome and transcriptome sequencing reads for each of these enzymes were normalized to the number of reads detected for the RNA polymerase *rpoBC*, a conserved single copy gene.

**Figure 5:**
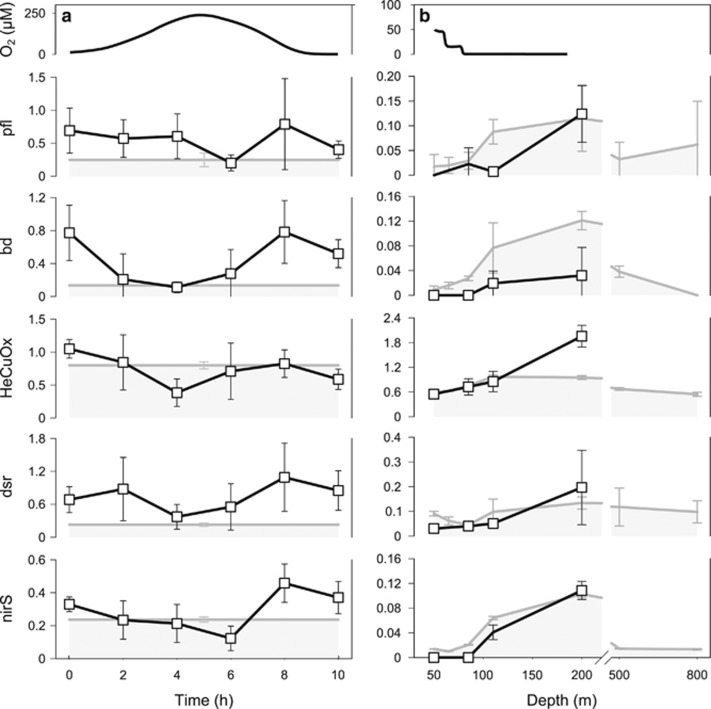
Normalized abundance (shaded areas) and activity (lines) of anaerobic genes in the environment. (**a**) Oxic marine sediment used as inoculum for the continuous cultures. (**b**) Chilean oxygen minimum zone (Canfield *et al.*, 2010). bd, *bd-*type terminal oxidase; dsr, dissimilatory sulfate reductase; HeCuOx, heme-copper oxidases; nirS, *cd-*type nitrite reductase; pfl, pyruvate formate lyase. Error bars indicate standard deviations.

Figure 5 shows both the normalized gene abundances and normalized transcriptional activity for the five functional genes in the temporal oxygen gradient of the intertidal flat (Figure 5a) and in the spatial oxygen gradient of the oxygen minimum zone (Figure 5b). The activity of *dsrAB* genes in the Chilean oxygen minimum zone was previously used to show that sulfate reduction occurred in the presence of nitrate (in combination with rate measurements, Canfield *et al.*, 2010). Activity of sulfate reduction genes was also detected under the denitrifying conditions of the intertidal flat. For both ecosystems, fermentation with acetate and formate as end products (inferred from the activity of pyruvate formate lyase, *pflAB*) occurred with oxygen or nitrate present in the bulk water.

## Discussion

The present study investigated how spatio-temporal chemical gradients shape microbial community structure at low (<20 μm) oxygen and nitrate concentrations. Two scenarios were considered. In the first scenario, the microbial redox tower was applied strictly. In the second scenario, all redox processes were allowed to proceed simultaneously.

Direct evidence for the parallel occurrence of unsorted redox processes consisted of the turnover of formate, succinate and reduced sulfur compounds, and the occurrence of denitrification in the presence of oxygen. This evidence was supported by the transcription and expression of functional genes associated with each of these processes by specific populations and by the presence of one abundant respiring (aerobic/denitrification) population (*Arcobacter*) that was unambiguously dependent on fermentation products and sulfide. For each abundant population, its metabolism, inferred *de novo* from genomics data, was completely consistent with literature data and available whole-genome sequences of cultivated relatives.

The observation that, in the absence of microaggregates, denitrification only occurred at high oxygen concentrations, was totally unexpected. The simultaneous transcription and expression of the *cbb*_3_ terminal oxidase and the nitrate, nitrite, nitric oxide and nitrous oxide reductases indicated that all complexes might compete for electrons in a branched respiratory chain (see also discussion below). The *cbb*_3_ terminal oxidase usually sustains microaerophilic metabolism (Pitcher and Watmough, 2004). Indeed, we showed inhibition at high oxygen concentrations and this might explain why, at oxygen concentrations above 0.15 mm, the denitrification enzymes became the prevalent electron sink. Because nitrous oxide reductase is the only intrinsically oxygen-sensitive enzyme of the denitrification pathway (Honisch and Zumft, 2003), nitrous oxide was the end product of denitrification at high oxygen concentration.

The direct evidence was supported by a stoichiometric model of microbial metabolism consisting of 21 reactions inferred from the measured gene activities and common bioenergetic constraints. With this model, we showed that both the experimentally observed low overall biomass yield and the relative abundances of the five populations were consistent with the observed gene expression patterns and our current understanding of bioenergetics. Several factors contributed to the low growth yield. First, the best carbon substrates were used for fermentation, instead of being assimilated by respiring populations. This lowered biomass yields, because fermentative metabolism conserves relatively little energy and because the bioenergetically more efficient respiring populations now needed to perform more work during the assimilation of fermentation products, compared with what would have been required to assimilate the best substrates directly. Second, the abundant *Vibrio* population consumed part of the oxygen using a cytochrome *bd* terminal oxidase. This oxidase has a high affinity for oxygen (low micromolar range), accepts electrons from quinols (bypassing respiratory complex III) and does not translocate protons (Jünemann, 1997). At a proton motive force of 180 mV, the bioenergetic efficiency of a respiratory chain terminated by a *bd* oxidase is only ~32%, compared to ~80% for a canonical respiratory chain with a complex III and terminated by a heme-copper-oxidase-based complex IV. Thus, use of cytochrome *bd* oxidases by *Vibrio* and *Desulfovibrio* populations compromised their biomass yield, and reduced the productivity of the microbial community as a whole. It is unknown whether the cytochrome *bd* terminal oxidase has a higher affinity for oxygen than the heme-copper *cbb*_*3*_ type terminal oxidases (Pitcher and Watmough, 2004) used by Rhodobacterales and *Arcobacter.* If this were the case, it would explain the accumulation of formate during the oxic part of the tidal cycle.

At this point, one might argue that the observed aggregation of cells in the chemostat created a second spatial compartment and that the localization of denitrification (during oxic conditions), fermentation and sulfate reduction to these aggregates could rescue the redox tower scenario. All populations were represented both by suspended and aggregated cells, so if this would be the case, it would mean that each population consisted of a mixture of phenotypically differentiated cells (for example, Schreiber *et al.*, 2016), with suspended cells performing a different redox process than aggregated cells. For denitrification, this interpretation was falsified experimentally by showing that suspended cells were still capable of denitrification and that aerobic respiration was inhibited in the presence of nitrate. The latter observation was most parsimoniously explained by competition for electrons between the terminal oxidase and the denitrification enzymes (see also discussion above), which meant that these enzymes must have been present together in the same (suspended) cells. Furthermore, stoichiometric modeling of the thermodynamically sorted scenario (reactions *a*–*c*) showed that this scenario would lead to a much higher biomass yield. A smaller portion of the carbon substrates would be respired and nitrite would never become a limiting substrate. Thus, the aggregates would be fully exposed to nitrite and the redox tower would predict that fermentation should not occur.

The combined activity of multiple redox processes within the same cells provided synergistic fitness benefits. For example, Rhodobacterales, *Vibrio* and *Arcobacter* combined denitrification with aerobic respiration. These populations bypassed the need for the bioenergetically costly turnover of enzyme systems. They could swiftly respond to the temporal fluctuation of the oxygen concentration without synthesizing new proteins. Furthermore, their hybrid respiratory chains even optimally prepared them for higher oxygen concentrations. *Vibrio* and *Arcobacter* protected oxygen-sensitive molecular machinery (nitrous oxide reductase, pyruvate formate lyase) by active respiration of oxygen. And even though *Vibrio*'s respiratory chain had low bioenergetic efficiency, respiration was still a much more efficient sink for excess electrons than resorting to mixed acid fermentation.

In oxygen-limited natural habitats, microbes experience continuous change and have three options: (i) differentiation of cells within each population (‘bet hedging', Schreiber *et al.*, 2016), (ii) continuously adapt by modulating gene expression via regulation or (iii) avoid regulation by stable expression of a set of genes that is adequate (but not optimal) for all conditions encountered. Here we mainly observed the latter ecological strategy. This increased resilience (more effective response to fluctuating conditions) at the expense of reduced productivity (biomass yield). The ecological success of fermentative populations, which outcompeted respiring populations for carbon substrates, showed that Zieglers principle better explained microbial community structure than Prigogine's theorem of the non-equilibrium steady state. Finally, our study provided strong evidence for the ecological importance of division of labor, both in carbon metabolism and in denitrification.

Future study will show to what extent these conclusions can be generalized. The presence and activity of a number of key functional genes of ‘braided' metabolism in two oxygen-limited habitats confirms and expands previous work (Canfield *et al.*, 2010; Ganesh *et al.*, 2015) and the model might thus at least be a useful template for the interpretation or experimental design of future studies of oxygen-limited natural ecosystems.
