# Supplementary figures and images for: Impacts of chemical gradients on microbial community structure
Source: ISME J. 2017 Jan 17;11(4):920–31. doi: 10.1038/ismej.2016.175 (PMC5363838; doi:10.1038/ismej.2016.175)

Supplementary Figure 2

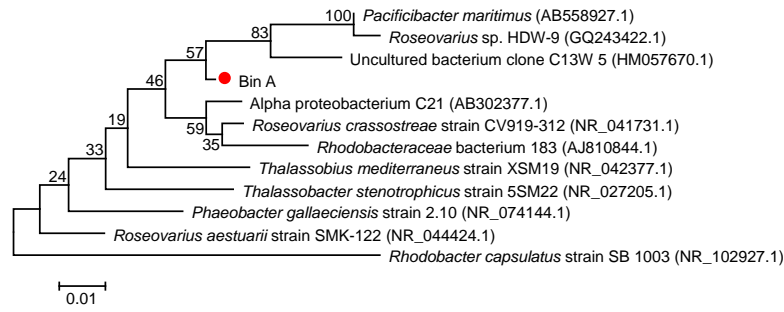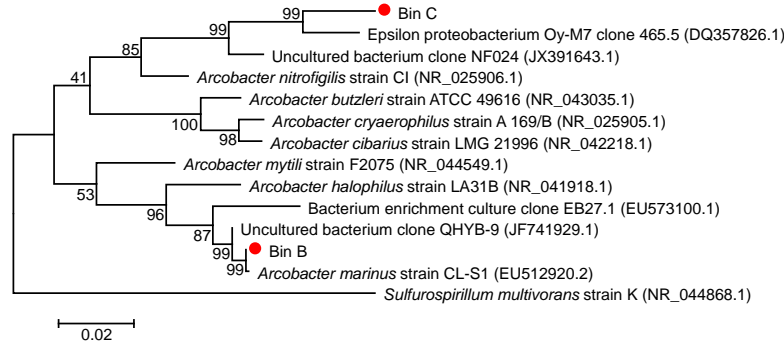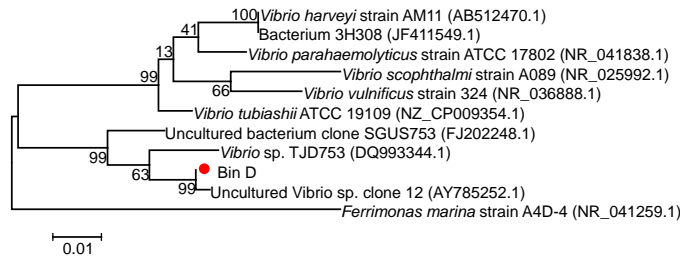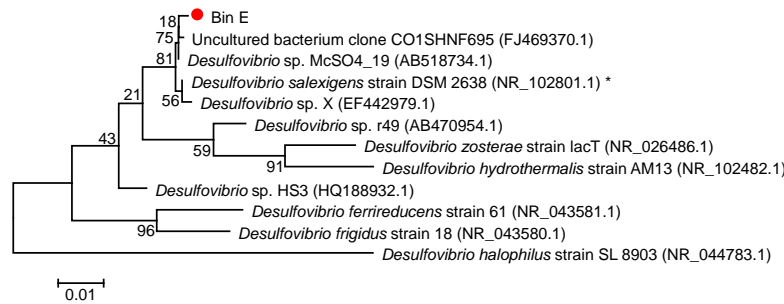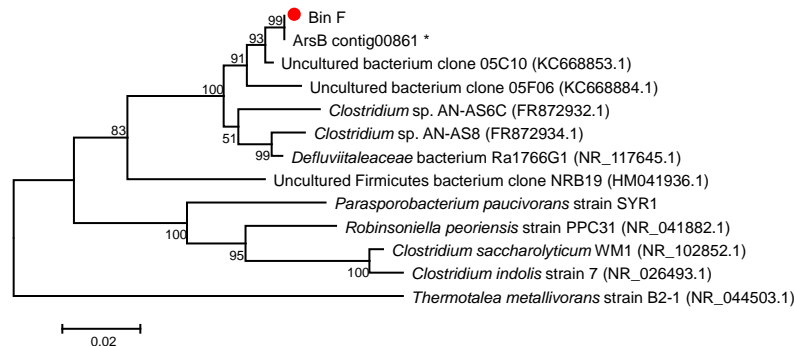

Supplement: Supplementary Figure 2 [file ismej2016175x2.pdf]
