# Supplementary material for: Impacts of chemical gradients on microbial community structure
Source: ISME J. 2017 Jan 17;11(4):920–31. doi: 10.1038/ismej.2016.175 (PMC5363838; doi:10.1038/ismej.2016.175)
Supplement: Supplementary Table 1 [file ismej2016175x3.pdf]

Supplementary Table 1. Probes and formamide concentrations used for fluorescence in situ hybridization (FISH). FISH was performed both with and without catalyzed reporter deposition.

Probe	Sequence (5′→3′)	Target organisms	FA (%)	Ref
EUB338 I	GCTGCCTCCCGTAGGAGT	Most Bacteria	35*,†	Aman et al, 1990
NON338	ACTCCTACGGGAGGCAGC	Control	35*,†	Wallner et al, 1993
GRB	GTCAGTATCGAGCCAGTGAG	Rhodobacter	25*	Eilers et al, 2000
GV	AGGCCACAACCTCCAAGTAG	Vibrio	30*	Eilers et al, 2000
ARC94	TGCGCCACTTAGCTGACA	Arcobacter	25†	Snaidr et al, 1997
ARC1430	TTAGCATCCCCGCTTCGA	Arcobacter	25†	Snaidr et al, 1997
DSV698 (& competitor)	GTTCCTCCAGATATCTACGG (GTTCCTCCAGATATCTACGC)	Some Desulfovibrio, Bilophila wadsworthia, and Lawsonia intracellularis	50*	Manz et al, 1998
CLO864	TTCCTCCTAATATCTACGCA	Clostridia	30*	Kraft et al, 2014
ALT1413	TTTGCATCCCACTCCCAT	Alteromonas, Colwellia	40†	Eilers et al, 2000
PS440LP	CCCTTCCTCCCAACTT	Pseudomonads	35†	Lenarts et al, 2007
PSA184	CCCCTTTGGTCCGTAGAC	Pseudoalteromonas, Colwellia	40†	Eilers et al, 2000

*) Formamide concentration in the CARD FISH hybridization buffer
†) Formamide concentration in the MONO FISH hybridization buffer
