# Supplementary material for: Impacts of chemical gradients on microbial community structure
Source: ISME J. 2017 Jan 17;11(4):920–31. doi: 10.1038/ismej.2016.175 (PMC5363838; doi:10.1038/ismej.2016.175)
Supplement: Supplementary Table 2 [file ismej2016175x4.pdf]

Supplementary Table 2. Abundance estimates and characteristics of metagenomic bins representing the five major clades of the selected microbial community and estimated biomass yields for all clades based on stoichiometric modelling. Bins A-D were obtained directly by assembly and tetranucleotide binning. For bins E and F assembly was generally unsuccessful and numbers are shown in parentheses and refer to genome data of closely related species (based on the assembled 16S rRNA genes shown in Supplementary Figure 2) that were also used as a template for mapping the transcriptome reads and for proteomics.

**Bin**	**Total**	**A**	**B**	**C**	**D**	**(E)**	**(F)**
Affiliation	-	Rhodo-bacterales	Arco-bacter	Arco-bacter	Vibrio-nales	*Desulfo-vibrio salexigens*	Firmicutes
Genome size (Mb)	17.9	3.86	3.29	2.38	5.19	(4.29)	(13.8)
Number of contigs (#)	20071	295	2230	4260	6844	(1)	(499)
N50 contig length (kb)	0.93	19.7	2.9	0.65	1.1	4.29	73.9
GC content (%)	35.4	57.5	26.6	29.1	43.2	47.1	33.7
Number of CSCGs (#)*	438	135	123	92	154	(132)	(291)
Number of tRNAs (#)	165	41	38	12	77	(89)	(137)
Bin name given by MetaWatt	-	L0,L1	L2,L6[†]	L5[†]	L3,L4	-	-
**Population abundances (%)**							
DNA (day 83)	58.7	34.7	14.4	0.7	4.9	0.5	3.6
DNA (day 90)	61.3	29.0	7.9	5.4	12.9	1.2	4.8
DNA (day 97)	65.6	41.9	14.3	2.7	6.2	0.5	0.0
RNA (oxic, day 84)[‡]	88.1	25.3	18.0	3.5	17.4	4.3	5.9
RNA (anoxic, day 84)[‡]	84.3	21.1	7.0	1.8	32.7	3.3	5.4
RNA (oxic, day 87)[‡]	82.8	24.4	24.8	8.3	8.4	2.9	7.8
RNA (anoxic, day 87)[‡]	80.4	23.4	15.4	12.4	10.2	4.6	8.2
DNA (normalized, average)	100	57 ± 10	20 ± 6	5 ± 4	13 ± 7	1.2 ± 0.7	5 ± 4
RNA (normalized, average)	100	28 ± 2	20 ± 9	8 ± 6	21 ± 13	5 ± 1	8 ± 2
Proteome (day 83)	100	43 ± 0	21 ± 0	4 ± 0	8 ± 0	8 ± 0	16 ± 0
FISH (day 83)	100	64 ± 7	13 ± 2		16 ± 2	3.8 ± 0.4	2 ± 0.2
16S tag sequencing (day 83)	91.6	41.9	32.2		5.2	0.9	11.4
Model predictions	100	48 ± 5	23 ± 2		18 ± 2	1 ± 0	10 ± 1
**Yield estimates (% of carbon assimilated, excluding storage materials)**							
Experimental (day 49-83)[§]	37 ± 8						
Model predictions[¶]	33	37	25	24	11	16	N/A
Model predictions (pure culture)[¶]	54						
**Biomass protein content (% w/w)**							
Experimental (day 49-83)	32 ± 9						
Model predictions[¶]	37						

*) Number of Conserved Single Copy Genes detected (out of a set of 139). Numbers higher than 139 may indicate the presence of DNA originating from more than a single population in the bin. Numbers

lower than 139 indicate the provisional genome sequence associated with the bin may be incomplete.

†) Cross binning between bins B and C was corrected based on the GC versus coverage plot using MetaWatt's manual bin editing features.

‡) Transcriptomes of day 87 were obtained directly from the culture; samples of day 84 were obtained from secondary anaerobic and aerobic batch incubations with filtered biomass from the culture (suspended cells).

§) Based on measurement of protein and particulate organic carbon concentration measurements in the culture

¶) Averages of estimates based on DNA, RNA, proteomes, 16S rRNA tag sequencing and FISH.

N/A, not applicable.
