# Supplementary material for: Impacts of chemical gradients on microbial community structure
Source: ISME J. 2017 Jan 17;11(4):920–31. doi: 10.1038/ismej.2016.175 (PMC5363838; doi:10.1038/ismej.2016.175)
Supplement: Supplementary Methods [file ismej2016175x5.doc]

**Supplementary methods**

**Stoichiometric modeling of metabolism*.*** Three overall reactions (Fig. 3a, *a-c*) were defined that described the metabolism of the thermodynamically sorted (redox tower) scenario. These reactions consist of the aerobic oxidation of the combined carbon sources (glucose, acetate, seven different aminoacids), the anaerobic oxidation of the combined carbon sources with nitrate and the anaerobic oxidation of the combined carbon sources with nitrite. Eighteen overall reactions (Fig. 3a, *d-v*) were defined that describe the main metabolisms inferred from the metagenomes, transcriptomes and proteomes (summarized in Fig. 3c). These reactions consist of various types of fermentation (*p-s, v*) and respiration (*d-o, t*). For the inference of these reactions we considered expression and transcription of genes encoding terminal oxidases, the denitrification pathway, respiratory complexes I-III, the citric acids cycle, glycolysis, Acetyl-CoA metabolism, hydrogenases, and transporters. Based on the inferred population genomes, each overall reaction was assigned to a specific population.

Each overall reaction was the sum of a catabolic reaction describing substrate conversion into products and an anabolic reaction describing assimilation (growth). For each overall reaction, the stoichiometry of the anabolic and the catabolic reactions was estimated according to Heijnen et al. (1992) and Kleerebezem and Van Loosdrecht (2010). These papers describe a generic method to estimate how many molecules of the energy source (*n*) need to be catabolized for each molecule of carbon source assimilated. This is done with the following equation:

*n* · ΔG_catabolic reaction_ = ΔG_anabolic reaction_ - ΔG_dissipated_

with *n* the number of catabolic reactions per anabolic reaction, ΔG_catabolic reaction_ the Gibbs free energy change of the catabolic reaction, ΔG_anabolic reaction_ the Gibbs free energy change of the anabolic reaction and ΔG_dissipated_ the amount of energy dissipated during anabolism. Basically, the latter is the energy needed to convert the carbon source into biomass. Heijnen et al. (1992) showed that that is a function of the number of carbon atoms and the degree of reaction of the carbon source. They also showed that using this equation, biomass yield could be predicted with reasonable accuracy for any metabolism. In the present paper, all Gibbs free energy changes were calculated for the actual conditions in the chemostat (pH 8.0, 25°C, substrate concentrations as described in the Results section)

To provide an example of this procedure, take overall reaction *t* (Figure 3a, sulfate reduction with H_2_ as the energy source and acetate as the carbon source). The catabolic reaction is:

SO_4_^2-^ + 4 H_2_ + H^+^ = HS^-^ + 4 H_2_O (with ΔG_catabolic reaction_ = -192 kJ/mol).

The anabolic reaction is:

0.59 C_2_H_3_O_2_^-^ + 0.2 NH_4_^+^ + 0.04 SO_4_^2-^ + 0.05 PO_4_^3-^ + 0.4 H^+^ = CH_1.8_O_0.5_N_0.2_S_0.04_P_0.05_ + 0.17 HCO_3_^-^ + 0.52 H_2_O (with ΔG_anabolic reaction_ = -22.7 kJ/mol).

The energy dissipation for assimilation of acetate according to Heijnen et al (1992) is 432 kJ/mol biomass produced.

This means that *n* = 2.4, so 2.4 catabolic reactions are needed for the each anabolic reaction, and leads to the following overall reaction:

0.59 C_2_H_3_O_2_^-^ + 0.2 NH_4_^+^ + 0.05 PO_4_^3-^ + 2.4 SO_4_^2-^ + 2.8 H^+^ + 9.5 H_2_ = 1 CH_1.8_O_0.5_N_0.2_S_0.04_P_0.05_ + 10 H_2_O + 0.17 HCO_3_^-^ + 2.4 HS^-^

In the present study, energy yields of respiratory catabolism were corrected based on the known degree of coupling that follows from biochemical characterization of the relevant enzyme complexes (e.g. for *bd* type terminal oxidases and NO-reductase).

Supplementary dataset S11 lists reactions *d-v* on rows 4-25. For each reaction, column E shows the predicted biomass yield (computed from the overall reactions). Columns G-AF show the stoichiometry of each molecule in each reaction. Columns AG-AM show that the reactions have valid stoichiometry, as all elements (as well as charge) are balanced.

One additional reaction (glycogen production, row 27) was added to explain continuous electron donor consumption with discontinuous electron acceptor supply (see also Fig. 1C). This reaction enabled the transfer of reducing equivalents from the anoxic to the oxic period of the cycle.

After the computation of all overall reactions was complete, we considered the mass balance for all carbon and nitrogen compounds, as well as oxygen, biomass and electrons over the continuous culture during one tidal cycle. For each compound, the consumption or production rate was estimated from the mass balances. Supplementary dataset S11 shows the experimentally observed production and consumption rates (mmol/day) of each molecule on line 29. Consumption is shown as negative values.

Next, we adjusted the rates of reactions *d-v* in column F until the modeled consumption (or production) rates of all molecules matched the experimentally observed values as best as possible. The outcome is shown on row 30. Row 31 shows the differences between the model predictions and the experimental observations. While adjusting the rates in column F, we also considered community structure. The overall biomass production rate for a population was computed from the rates of all reactions assigned to that population and this value was used to predict the relative abundance of that populations. The relative abundances predicted by the model were compared to the experimentally observed abundances (based on FISH, transcripts, proteomes and metagenomes, see Supplementary Table 2).

This way, the model explained the experimentally observed conversion rates for all compounds, as well as the observed community structure, with two exceptions (shown in red on row 31). First, the predicted biomass production rate was ~35% higher than observed experimentally. Second, the predicted production rate of elemental sulfur was four times higher than measured. The latter can easily be explained by precipitation of elemental sulfur on the culture vessel walls (which was not quantified). For the former, this might be explained by limitations of experimental methods to quantify biomass, overestimation of biomass yields by the method of Heijnen et al. (1992) and/or by other ecological processes such as viral predation.

Finally, we also solved the model for reactions *a-c* (describing a microbial redox tower scenario) by adjusting reaction rates in column F for rows 36-42. These reactions could not be reconciled with the experimental results. The redox tower model predicted a much too high biomass yield and predicted that not all the provided nitrite would be consumed with the electron donors provided.

The width of the arrows in Fig. 3C is proportional to the predicted conversion rates of reactions *d-v* (plus glycogen production) in Supplementary dataset S11 and shows the inferred flows of the elements through the microbial community selected in the chemostat.
